# Green Fabrication of Supported Platinum Nanoparticles by Supercritical CO_2_ Deposition

**DOI:** 10.3390/ma11122587

**Published:** 2018-12-18

**Authors:** Ying-Liang Chen, Cheng-Hsien Tsai, Mei-Yin Chen, Yi-Chieh Lai

**Affiliations:** 1 Department of Environmental Engineering, National Cheng Kung University, Tainan 70101, Taiwan; roy.yl.chen@gmail.com; 2 Department of Chemical and Materials Engineering, National Kaohsiung University of Science and Technology, Kaohsiung 80778, Taiwan; chtsai@nkust.edu.tw; 3 Department of Chemistry, Chung Yuan Christian University, Chung-Li 32023, Taiwan; h577577@gmail.com; 4 Department of Safety, Health and Environmental Engineering, National Kaohsiung University of Science and Technology, Kaohsiung 82445, Taiwan

**Keywords:** Supercritical deposition, platinum, carbon dioxide, factorial design

## Abstract

Pt nanoparticles were successfully deposited on uncatalyzed carbon paper by the supercritical CO_2_ deposition (SCD) method using platinum (II) acetylacetonate as a precursor followed by thermal conversion. A full 2^4^ factorial design (four factors, each with two levels) was used to investigate the main effect of four factors (deposition temperature, deposition time, reduction temperature, and reduction time) and the interaction effects between them. The morphological structures and surface properties of the Pt/carbon paper composite were analyzed by X-ray diffraction (XRD), scanning electron microscope (SEM)/energy-dispersive X-ray spectroscopy analyzer (EDS), and high-resolution transmission electron microscopy (HR-TEM). The results of the 2^4^ factorial design showed that Pt loading on the substrate correlated significantly with deposition time, while Pt aggregation slightly increased with the thermal reduction temperature. Data obtained from both XRD and HR-TEM were in good agreement and showed that Pt nanoparticles were homogeneously dispersed on the substrate with diameters of 7.2–8.7 nm.

## 1. Introduction

Supported noble-metal nanoparticles have recently been attracting attention in the field of microelectronics, optics, and catalytic applications due to their high activity and stability [1,2,3]. Pt nanoparticles are the most effective catalyst known for oxygen reduction, which is widely used for fuel cells [4,5]. Since fuel cells play a significant role in the generation of power, much attention has been given to the preparation of high-performance Pt-based nanoparticles for low temperature fuel cells [4,5,6]. These catalysts rely on various conventional techniques such as wet impregnation, spraying, sol-gel, chemical vapor deposition and electro-deposition [7,8]. Among these techniques, several challenges remain, including particle dimensions (size and distribution) and metal loading. Although the precise shapes and sizes of Pt nanoparticles can be fabricated by the modified version of polyol methods [9,10], the use of toxic and hazardous reagents and solvents in the fabrication are considered an important environmental problem. 

Interest in supercritical fluids and their potential use for metal nanoparticle preparations has significantly increased over the past decade. Supercritical CO_2_ (scCO_2_) is considered a green solvent for nanoparticle preparations following the deposition process because of its environmentally benign, non-toxic, non-polluting, and recoverable characteristics [11,12,13]. For Pt nanoparticles prepared by supercritical CO_2_ deposition (SCD), the appropriate Pt loading and catalyst structures are determined by the following parameters: types of substrate and precursor; deposition pressure; temperature and time; and, conversion method [3]. However, little information is available on the main and interaction effects of the SCD factors on particle characteristics (i.e., loading, size, and microstructures).

The purpose of this study was to determine the effects of the SCD conditions on the supported Pt characteristics. A full 2^4^ factorial design was used to evaluate the main effects as well as the interaction effects of the parameters during SCD with a 95% confidence limit. The morphological structures and surface properties of the Pt/carbon paper composite were analyzed by X-ray diffraction (XRD), scanning electron microscope (SEM)/energy-dispersive X-ray spectroscopy analyzer (EDS), and high-resolution transmission electron microscopy (HR-TEM). 

## 2. Materials and Methods

### 2.1. Preparation of Pt Nanoparticles via Supercritical CO_2_ Deposition (SCD)

**Figure 1:**
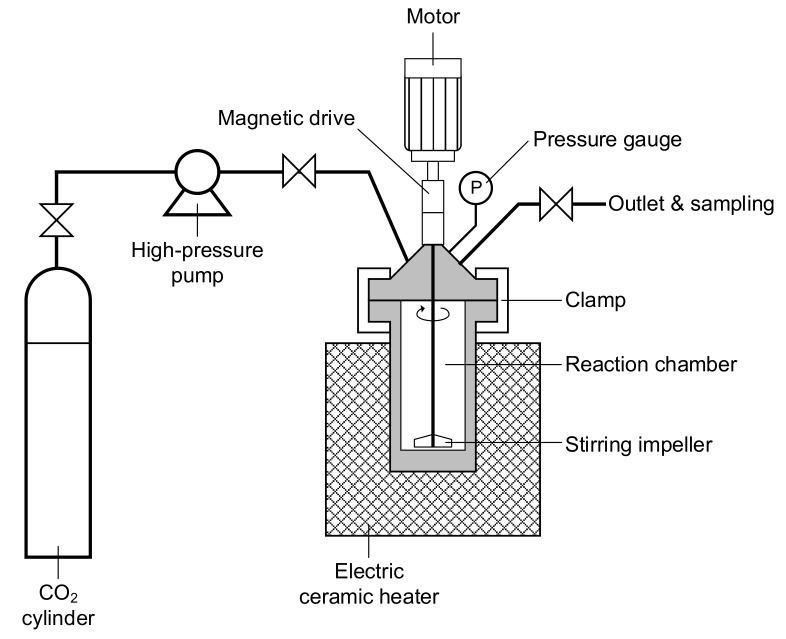
Schematic of the experimental setup.

The SCD experiment was performed in a high-pressure reaction chamber. A schematic view of the experimental setup is provided in Figure 1. The deposition apparatus consisted of a 100 mL stainless steel reaction chamber (SS-316, Amar Equipment, Ltd., Mumbai, India) equipped with a magnetic stirring bar, high-pressure pump, heating unit, pressure gauge, and inlet and outlet valves. The supercritical fluid used in this study was CO_2_ gas (purity > 99.99%; Jing De Gases, Ltd., Kaohsiung, Taiwan), and a high-pressure pump (Model PM-10000B, Taiwan Supercritical Technology, Ltd., Changhua County, Taiwan) was used to pressurize the CO_2_ to the desired level. The high-pressure reaction chamber was then sealed with a stainless-steel clamp (Amar Equipment, Ltd., Mumbai, India) then heated to the desired temperature by a circulating heater/cooler (Amar Equipment, Ltd., Mumbai, India). 

**Figure 2:**
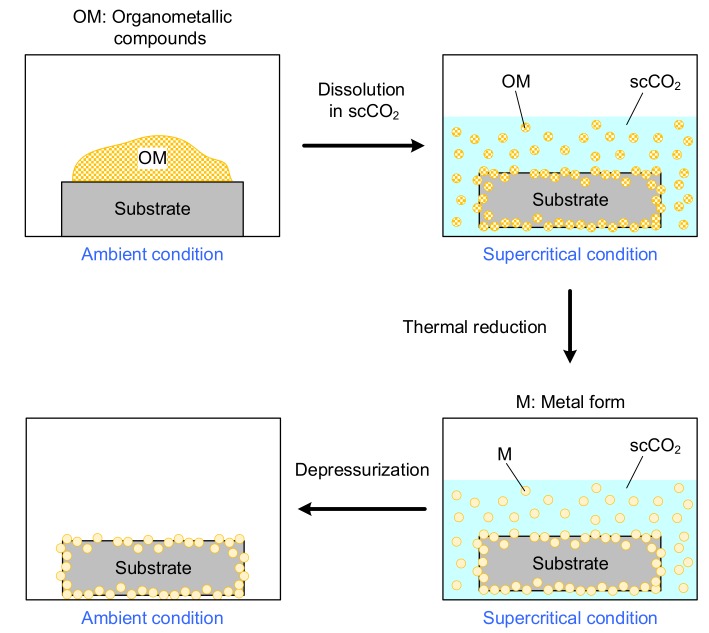
Schematic representation of the supercritical CO_2_ deposition (SCD).

The SCD process involved three main steps: (1) dissolution of a metallic precursor in scCO_2_ under a given condition; (2) molecular adsorption of the precursor on the support; and, (3) decomposition of the precursor to its metal form. In general, a metallic precursor can be converted to its metal form using different methods, including: (1) chemical reduction in scCO_2_ with a reducing agent (hydrogen); (2) thermal reduction in scCO_2_; (3) thermal reduction in an inert atmosphere; or, (4) chemical reduction with hydrogen at ambient pressure [1,2,3]. In this study, the precursor was reduced thermally in scCO_2_, as shown in Figure 2.

For each SCD experiment, a known amount of platinum (II) acetylacetonate (Pt(II)(acac)_2_; 98%) (Acros Organics, Fukuoka, Japan) precursor was placed into the stainless-steel reaction chamber, together with the activated carbon paper (without catalyst) (CeTech™, Taichung, Taiwan), for a projected active area of 5 cm^2^. The amount of Pt(II)(acac)_2_, calculated from the solubility in scCO_2_, was 10 times the saturating amount at the supercritical condition and, therefore, Pt(II)(acac)_2_ was fully soluble in scCO_2_ [14]. A piece of carbon paper (CeTech™, Taichung, Taiwan), Pt(II)(acac)_2_, and methanol (Acros Organics, Fukuoka, Japan) were placed in a high-pressure vessel (Amar Equipment, Ltd., Mumbai, India), which was slowly pressurized with CO_2_ (99.998%) at the given pressure (10.3 MPa) and heated to the selected temperature for durations varying from 2 to 6 h. Note that methanol was used as a co-solvent to enhance the solubility of the precursor in the scCO_2_ [3,15]. The impregnated precursor was then reduced thermally at 200 and 250°C for 2–4 h under the supercritical environment. Once the reduction process was finished, the pressure was slowly released and the chamber cooled to room temperature. The Pt/carbon paper composite was then removed from the chamber and stored for further analysis. 

### 2.2. Full 2^4^ Factorial Design

In order to gain more insight into the significance of the factors and their interaction on the Pt loading via SCD, experiments were designed. In general, a 2^n^ factorial design is the most common type of factorial design as it applies two levels and thereby reduces the number of experimental conditions [16,17]. In this manner, the reliability and efficiency of the obtained data can be improved through factorial design experiments. 

In this study, a full 2^4^ factorial design (four factors, each with two levels) with a total of 16 experiments was used to investigate the main effect of four factors (deposition temperature and time, as well as reduction temperature and time) and the interaction effects between them. The high (+1) and low (−1) levels of the factors were selected based on previous experiments and the scientific literature [2,3,13]. The experiments were consistent with theories of factorial design, whereby all levels of each factor are combined with those of every other factor. Table 1 shows the factors and levels investigated in the factorial design. The full 2^4^ factorial design matrix and experimental responses (i.e., Pt loading) are presented in Table 2. The results of the 2^4^ factorial design were analyzed by analysis of variance (ANOVA, Minitab 18 statistical software (Minitab, Inc., Pennsylvania, USA)) and differences were considered as significant based on the P value with > 5% confidence level. 

### 2.3. Characterization

The Pt loading of the Pt/carbon paper composite was determined by inductively coupled plasma mass spectrometry (ICP-MS) (7500a; Agilent, California, USA) after acid digestion. This was achieved by assuming that the entire Pt in the adsorbed precursor was reduced to elemental Pt without volatilization during thermal reduction. The morphology of the Pt/carbon paper samples was evaluated using an SEM (JSM-7600F; JEOL Ltd., Tokyo, Japan) with the EDS (JEOL Ltd., Tokyo, Japan). The Pt/carbon paper samples were also examined using a HR-TEM (CM-200; Philips, Amsterdam, Netherlands) to determine their sizes, morphologies, and microstructures. For SEM analysis, the Pt/carbon paper composite was fixed on a piece of double-sided adhesive copper tape. For HR-TEM analysis, the Pt nanoparticles were dispersed in methanol and then placed on a copper grid. The crystal structure of the as-prepared Pt nanoparticles was determined using the XRD (X’Pert PRO MPD; Philips, Amsterdam, Netherlands) with CuKa radiation that scanned from 20 to 90 °C (2θ).

## 3. Results and Discussion

### 3.1. Analysis of 2^4^ Factorial Design

As presented in Table 2, the Pt loading on the Pt/carbon paper composite ranged from 0.122 to 0.265 mg/cm^2^, with the maximum amount of Pt loading performed in scCO_2_ at 80 °C and 10.3 MPa for 6h then reduced thermally at 250 °C for 2 h. The main and interaction effects of the 2^4^ factorial design were determined using the General Linear Model of Minitab 18 statistical software. The ANOVA results for the factorial model are presented in Table 3. The p-value is defined as the smallest level of significance that results in the rejection of the null hypothesis, and is a useful tool in statistical analysis to indicate whether the result of the statistical hypothesis test is significant or not [16]. In general, a smaller p-value implies stronger evidence against the null hypothesis [17,18]. It was found that the p-values for the deposition time (DH) and reduction temperature (RT) factors were lower than 0.05 (95% confidence interval) for the response “Pt loading”, hence, these two factors had statistically significant effects (p < 0.05) on the response. As for the p-values of the two-factor interactions, all values were higher than 0.05, indicating that all interactions were not significant. In addition, an increased sum of squares (SS) value indicates the significance of the corresponding factor [19]. 

**Figure 3:**
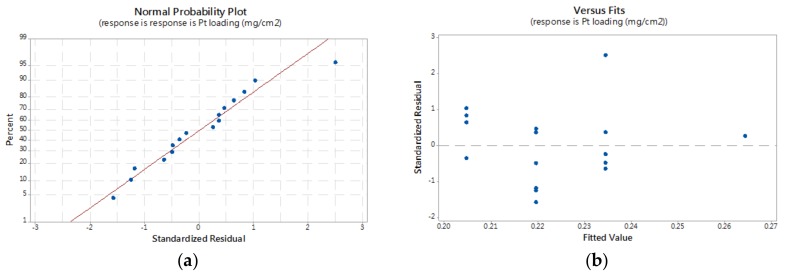
Residual plots in the regression analysis: (**a**) normal probability plot and (**b**) residuals versus fit of residuals.

The validity of the model was found using a normal probability plot of the residuals (Figure 3a) and the plot of residual versus the fit of the residuals (Figure 3b). The normal plot of the residuals was used to verify the normality of the treatment data. The absence of a straight line in Figure 3a resulted in a normal distribution of residual values. All points were found to fall in the range of +2 to −2, except for one point (run number 16), as seen in Figure 3b. Since no pattern was found in the plot, the constant variance assumption in the model was satisfied. Accordingly, the assumptions for both normality and constant variance of the model were met. 

### 3.2. Main and Interaction Effects

**Figure 4:**
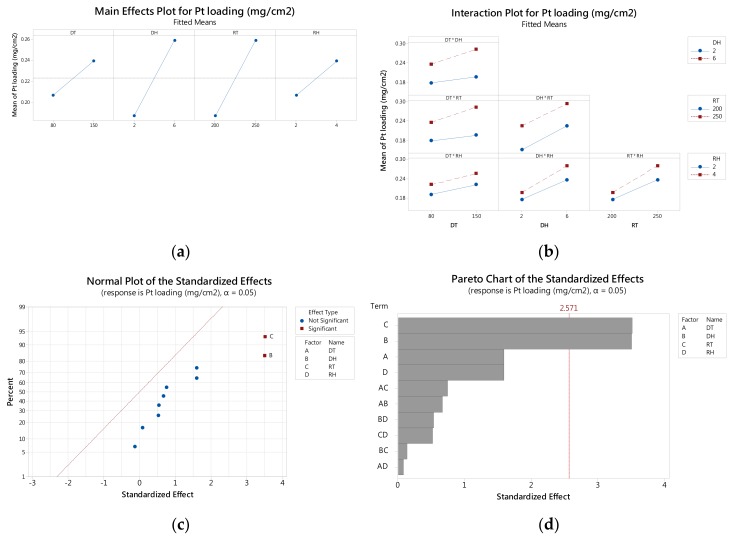
The factorial design plots of Pt loading on the Pt/carbon paper composite prepared by SCD: (**a**) main effects plot of response; (**b**) interaction plot of response; (**c**) normal plot of the standardized effect; and (**d**) Pareto chart of the standardized effect.

The main effects plot, as seen in Figure 4a, reveals variations between the high and low values of each factor. When the slope is positive, the Pt loading is increasing by a corresponding factor. In this study, the higher slope values of DH and RT indicate that their effects were the most significant. Furthermore, the parallel lines in Figure 4b indicate that there are no apparent interactions between factors. The normal probability plot (Figure 4c) was used to identify real effects [20]. Points (denoted as squares) located far away from the fitted line were considered significant. In this study, DH and RT, the main effects, were considered to have a significant effect on Pt loading, which is also confirmed in Figure 4a. A Pareto plot, as seen in Figure 4d, is typically applied to identify the significance of the factors and their interactions [19], which in this study was set at a 95% confidence interval. The values positioned to the right of the reference line (2.571) were considered significant factors. These results concur with the analysis of main effects, presented in Figure 4a. Accordingly, based on the factorial-design results shown in this study, Pt loading on the Pt/carbon paper composite fabricated by SCD can be enhanced by increasing the deposition time and reduction temperature.

### 3.3. Characterization of Pt/Carbon Paper Composites

**Figure 5:**
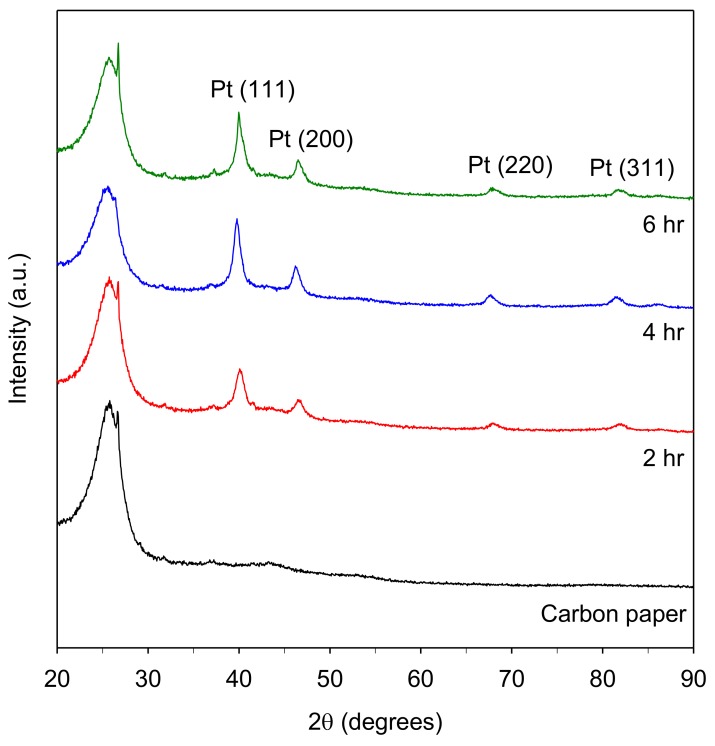
XRD patterns of Pt-deposited carbon paper fabricated by SCD at 80 °C and various time intervals by thermal reduction at 250 °C for 2 h.

Figure 5 presents the XRD patterns for the fabricated Pt/carbon paper samples. Four crystalline peaks corresponding to Pt (1 1 1), Pt (2 0 0), Pt (2 2 0), and Pt (3 1 1) were obtained, indicating that Pt was in its metallic form in all samples. It is concluded that the Pt(II)(acac)_2_ can be converted to its metal form at 250 °C. These findings are consistent with the results obtained from various supported Pt nanoparticles fabricated using SCD [2,21,22]. Moreover, the intensity of the Pt (1 1 1) peak was substantially greater than the others, revealing that the Pt crystal growth was predominately in the (1 1 1) direction. The mean size of the Pt (1 1 1) grains can be calculated using Scherrer’s formula, as presented in our previous study [6]. The calculated Scherrer crystallite size for samples with deposition times of 2, 4, and 6 h were 7.5, 7.9, and 8.4 nm, respectively, thereby demonstrating slight increases with increasing deposition time. 

**Figure 6:**
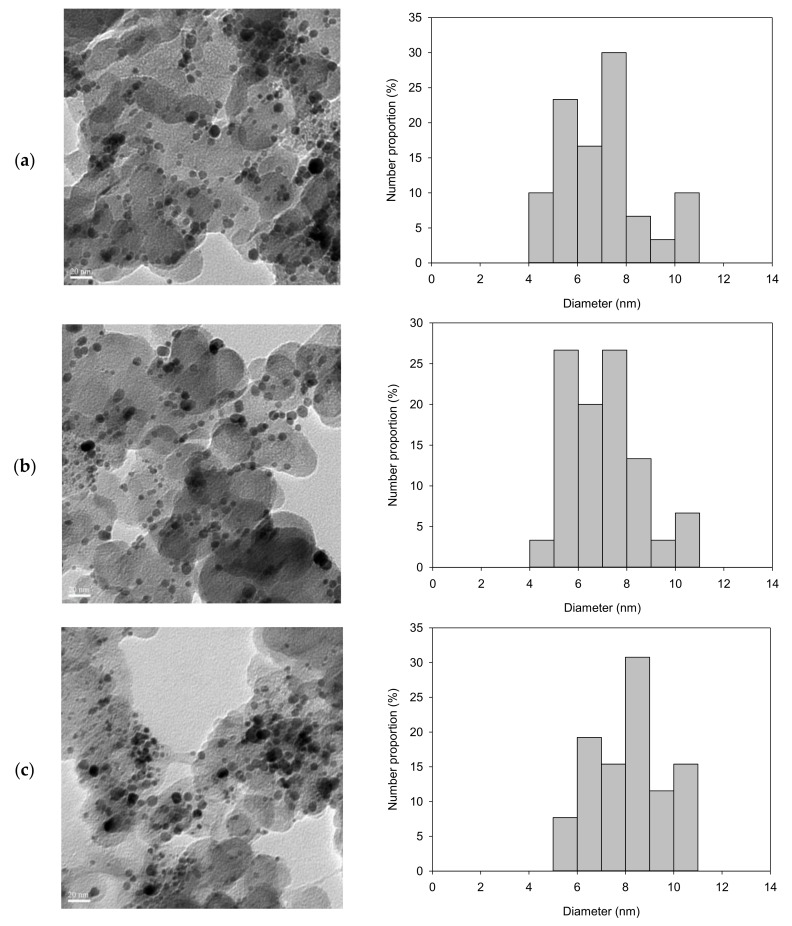
HR-TEM images and Pt particle-size distribution fabricated by SCD at 80 °C for different time durations: (**a**) 2 h; (**b**) 4 h; and, (**c**) 6 h.

The HR-TEM images and Pt particle size distribution on the Pt/carbon paper samples fabricated with different deposition times are given in Figure 6. Particle size distributions were obtained based on 100 particles on each sample randomly selected from the HR-TEM images. The results showed that the Pt nanoparticles were dispersed homogenously on the surface of the carbon paper. The mean particle size for samples with deposition times of 2, 4, and 6 h were 7.2, 8.4, and 8.7 nm, respectively, which concurs with the crystallite size obtained by XRD analysis (7.5–8.4 nm). Similarly, increasing the deposition time resulted in an increase in the Pt particle size of the Pt/carbon paper samples due to the aggregation of the Pt particles during SCD [6,10,21,22]. 

**Figure 7:**
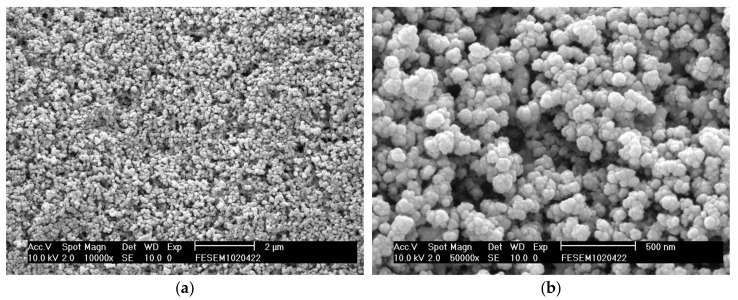

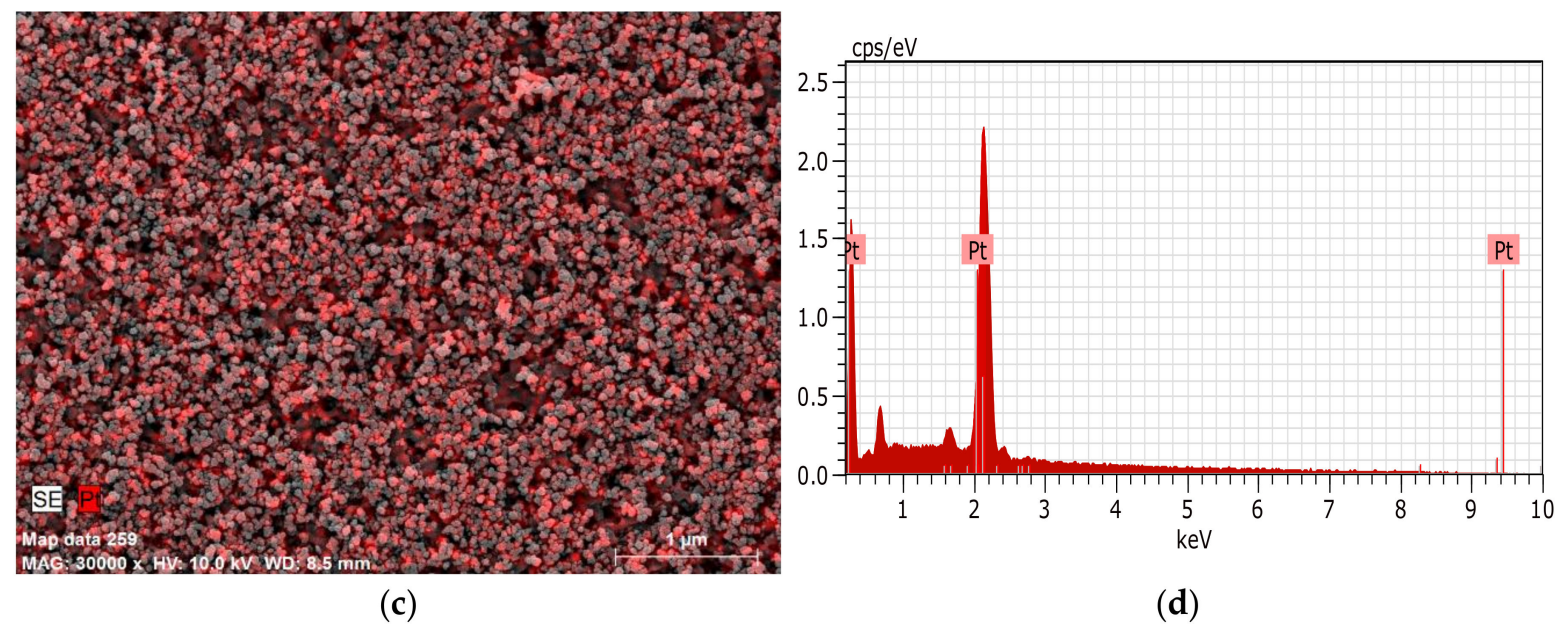
(**a**) SEM photograph (×10000), (**b**) SEM photograph (×50000), (**c**) EDX mapping image, and (**d**) EDX spectrum of supported Pt nanoparticles fabricated by SCD at 80 °C and 6 h.

Based on the analysis of variance and the factorial design of experiments, the optimal parameters for the Pt/carbon paper (with Pt loading of 0.265 mg/cm^2^) fabricated using SCD are listed as follows: deposition temperature = 80 °C; deposition time = 6 h; reduction temperature = 250 °C; and reduction time = 2 h. Figure 7 displays the top view SEM and EDX images of the above-mentioned sample. Figure 7a,b clearly indicate that the Pt particles were homogeneously dispersed on the carbon particles as well as inside the porous structure of the carbon paper. Analysis using EDX spectrometers (Figure 7c,d) confirmed the presence of the elemental Pt signal of the Pt/carbon paper samples, thereby confirming that Pt nanoparticles were successfully deposited via the SCD method. 

## 4. Conclusions

Pt nanoparticles were successfully deposited on non-catalyzed carbon paper by the SCD method using platinum (II) acetylacetonate as a precursor. A 2^4^ factorial design was applied to investigate the influence of parameters (deposition temperature and time, as well as reduction temperature and time) on the Pt loading of the Pt/carbon paper composite. The results showed that Pt loading on the substrate correlates significantly with deposition time, while Pt aggregation increased with increases in the thermal reduction temperature. Data obtained from both XRD and HR-TEM were in good agreement and showed that Pt was homogeneously dispersed on the substrate with diameters in the range of 7.2–8.7 nm. The optimal parameters for the Pt/carbon paper (with Pt loading of 0.265 mg/cm^2^) fabricated using SCD were found to be 80 °C for 6 h, followed by thermal reduction at 250 °C for 2 h, with the addition of methanol as a co-solvent. Overall, the properties of the supported metallic nanoparticles such as loading, size, distribution, and morphology depend closely on the conditions of the SCD method (type of substrate, precursor, temperature, time, conversion method, etc.). The results from this study could contribute to the development of commercial applications for SCD, however, further investigations of the obtained metallic nanoparticles properties (e.g., catalytic activity, and electrochemical performance) are needed.

## Figures and Tables

**Table 1:** Summary of the factors and levels included in the full 2^4^ factorial design.

Factors	Levels	
	Low(−1)	High(+1)
Deposition Temperature (DT)	80 °C	150 °C
Deposition Time (DH)	2 h	6 h
Reduction Temperature (RT)	200 °C	250 °C
Reduction Time (RH)	2 h	4 h

**Table 2:** Full 2^4^ factorial design matrix and experimental responses.

Run	Factor Combination	Factors				Pt Loading (mg/cm^2^)
		Deposition Temperature (A)	Deposition Time (B)	Reduction Temperature (C)	Reduction Time (D)	
1	1	−1	−1	−1	−1	0.122
2	A	+1	−1	−1	−1	0.147
3	B	−1	+1	−1	−1	0.183
4	AB	+1	+1	−1	−1	0.253
5	C	−1	−1	+1	−1	0.196
6	AC	+1	−1	+1	−1	0.242
7	BC	−1	+1	+1	−1	0.265
8	ABC	+1	+1	+1	−1	0.248
9	D	−1	−1	−1	+1	0.189
10	AD	+1	−1	−1	+1	0.142
11	BD	−1	+1	−1	+1	0.220
12	ABD	+1	+1	−1	+1	0.242

**Table 3:** Results of the analysis of variance (ANOVA) for Pt loading on the Pt/carbon paper composite prepared by the SCD process.

Source	Degree of Freedom (DF)	Adjust Sum of Squares (SS)	Adjust Mean Squares (MS)	F-Value	P-Value
DT	1	0.00424	0.00424	2.53	0.173
DH	1	0.020606	0.020606	12.29	0.017
RT	1	0.020696	0.020696	12.35	0.017
RH	1	0.004234	0.004234	2.53	0.173
DT*DH	1	0.000752	0.000752	0.45	0.533
DT*RT	1	0.000931	0.000931	0.56	0.490
DT*RH	1	0.000011	0.000011	0.01	0.939
DH*RT	1	0.000032	0.000032	0.02	0.896
DH*RH	1	0.000486	0.000486	0.29	0.613
RT*RH	1	0.000446	0.000446	0.27	0.628
Error	5	0.008381	0.001676		
Total	15	0.060814			
S = 0.0409422; R^2^ = 86.22%					

## References

[1] Türk M., Erkey C. (2018). Synthesis of supported nanoparticles in supercritical fluids by supercritical fluid reactive deposition: Current state, further perspectives and needs. J. Supercrit. Fluids.

[2] Sánchez-Miguel E., Tenorio M.J., Morère J., Cabañas A. (2017). Green preparation of PtRu and PtCu/SBA-15 catalysts using supercritical CO_2_. J. CO_2_ Util..

[3] Bozbag S.E., Erkey C. (2015). Supercritical deposition: Current status and perspectives for the preparation of supported metal nanostructures. J. Supercrit. Fluids.

[4] Huang K.L., Lai Y.C., Tsai C.H. (2006). Effects of sputtering parameters on the performance of electrodes fabricated for proton exchange membrane fuel cells. J. Power Sources.

[5] Sui S., Wang X., Zhou X., Su Y., Riffat S., Liu C. (2017). A comprehensive review of Pt electr Catalysts for the oxygen reduction reaction: Nanostructure, activity, mechanism and carbon support in PEM fuel cells. J. Mater. Chem. A.

[6] Lai Y.C., Huang K.L., Tsai C.H., Lee W.J., Chen Y.L. (2012). Sputtered Pt loadings of membrane electrode assemblies in proton exchange membrane fuel cells. Int. J. Energy Res..

[7] Firtina I., Güner S., Albostan A. (2010). Preparation and characterization of membrane electrode assembly (MEA) for PEMFC. Int. J. Energy Res..

[8] Yu W., Porosoff M.D., Chen J.G. (2012). Review of Pt-based bimetallic catalysis: from model surfaces to supported catalysts. Chem. Rev..

[9] Sápi A., Varga A., Samu G.F., Dobó D.G., Juhász K.L., Takács B., Varga E., Kukovecz Á., Kónya Z., Janáky C. (2017). Photoelectr chemistry by design: Tailoring the nanoscale structure of Pt/NiO composites leads to enhanced photoelectr chemical hydrogen evolution performance. J. Phys. Chem. C.

[10] Sápi A., Halasi G., Kiss J., Dobó D.G., Juhász K.L., Kolcsár V.J., Ferencz Z., Vári G., Matolin V., Erdőhelyi A. (2018). In Situ DRIFTS and NAP-XPS Exploration of the Complexity of CO_2_ Hydrogenation over Size-Controlled Pt Nanoparticles Supported on Mesoporous NiO. J. Phys. Chem. C.

[11] Saquing C.D., Kang D., Aindow M., Erkey C. (2005). Investigation of the supercritical deposition of platinum nanoparticles into carbon aerogel. Microporous Mesoporous Mater..

[12] Aspromonte S., Sastre A., Boix A., Cero M.J.C., Alonso E. (2016). Optimization and modelling of the supercritical CO_2_ deposition of Co_x_O_y_ nanoparticles in MCM41. J. Supercrit. Fluids.

[13] Oztuna F.E.S., Barim S.B., Bozbag S.E., Yu H.B., Aindow M., Unal U., Erkey C. (2017). Graphene aerogel supported pt electro Catalysts for oxygen reduction reaction by supercritical deposition. Electr Chim. Acta.

[14] Türk M., Crone M., Upper G. (2011). Effect of gas pressure on the phase behaviour of organometallic compounds. J. Supercrit. Fluids.

[15] Chen A.Z., Wang G.Y., Wang S.B., Feng J.G., Liu Y.G., Kang Y.Q. (2012). Preparation of poly-(methyl vinyl ether-co-maleic anhydride) nanoparticles by solution-enhanced dispersion by supercritical CO_2_. Materials.

[16] Montgomery D.C. (1991). Design and Analysis of Experiments.

[17] Berger P.D., Maurer R.E. (2002). Experimental Design.

[18] Sterne J.A.C., Smith G.D. (2001). Sifting the evidence—What’s wrong with significance tests?. Phys. Therapy.

[19] Farooq S., Saeed A., Sharif M., Hussain J., Mabood F., Iftekhar M. (2017). Pr Cess optimization studies of crystal violet dye adsorption onto novel, mixed metal Ni_0.5_Co_0.5_Fe_2_O_4_ ferrospinel nanoparticles using factorial design. J. Water Pr Cess Eng..

[20] Camacho L.M., Fox J.A., Ajedegba J.O. (2017). Optimization of electrodialysis metathesis (EDM) desalination using factorial design methodology. Desalination.

[21] Bozbag S.E., Yasar N.S., Zhang L.C., Aindow M., Erkey C. (2011). Absorption of Pt(cod)me_2_ onto organic aerogels from supercritical solutions for the synthesis of supported platinum nanoparticles. J. Supercrit. Fluids.

[22] Barım Ş.B., Bayrakçeken A., Bozbağ S.E., Zhang L., Kızılel R., Aindow M., Erkey C. (2017). Control of average particle size of carbon aerogel supported platinum nanoparticles by supercritical deposition. Microporous Mesoporous Mater..

